# Ventilatory failure following active humidification of a retained HMEF in an intubated infant: a case report

**DOI:** 10.3389/fsurg.2026.1837176

**Published:** 2026-05-13

**Authors:** Hengjing Zou, Guangyi Lai, Yanping Lu, Shan Ou

**Affiliations:** Center of Anesthesiology and Surgery, Chengdu Integrated TCM&Western Medicine Hospital, Chengdu, China

**Keywords:** active humidification, apparatus dead space, case report, heat and moisture exchanger filter, hypercapnia, pediatric anesthesia

## Abstract

**Background:**

Heat and moisture exchanger filters (HMEFs) provide passive humidification and microbial filtration during pediatric anesthesia, but their apparatus dead space and flow resistance—trivial in adults—may represent a clinically important fraction of tidal volume in infants. Progressive moisture saturation further increases resistance, and simultaneous use with an active heated humidifier can precipitate circuit occlusion.

**Case presentation:**

A 13-month-old boy undergoing ophthalmologic surgery developed wheezing, progressively rising peak inspiratory pressure (PIP), and hypercapnia (PaCO_2_ 81.5 mmHg) after a difficult intubation. Presumed bronchospasm was treated with salbutamol, sevoflurane escalation, epinephrine, hydrocortisone, and magnesium, producing only partial improvement. He was transferred intubated to the ICU with the same breathing circuit and HMEF *in situ*. Within approximately 10 min of connection to a ventilator equipped with active heated humidification, PIP rose to 40 cmH_2_O, delivered tidal volume fell from 90 mL to 50 mL, and PaCO_2_ rose to 119.5 mmHg. The HMEF was visibly saturated; its immediate removal produced prompt resolution of pressure, volume, wheeze, and blood gases.

**Conclusions:**

An unremoved HMEF exposed to active heated humidification can cause fulminant ventilatory failure in infants. HMEF dead space, saturation status, and humidifier compatibility must be explicitly verified before and during every pediatric perioperative transfer.

## 1 Introduction

Heat and moisture exchanger (HME) and heat and moisture exchanger filter (HMEF) are widely used in anesthesia and intensive care to provide passive humidification. The HMEF incorporates an added microbial filtration function, making it more widely used in anesthesia breathing circuits. However, these devices also add resistance and apparatus dead space, both of which are proportionally more significant in infants and small children than in older patients (1). Kwon demonstrated that a pediatric HME with an internal volume of 22 mL significantly increased PaCO_2_ in otherwise healthy children during anesthesia, and that the effect was greater in younger and lighter patients (2). In small children, even modest increases in apparatus dead space may produce disproportionate increases in PaCO_2_ or require substantial increases in minute ventilation to maintain normocapnia (3). A pediatric case report has previously described severe hypercapnia caused by dead space from an HMEF (4), and case reports in critically ill patients describe rising peak inspiratory pressures and ventilatory compromise from HME obstruction or saturation (5). Moreover, simultaneous use of an HME and active heated humidification can precipitate critical airway occlusion (6).

We report an infant with apparent refractory bronchospasm in whom the clinical course was later shown to have a major HMEF-related component culminating in fulminant ventilatory failure minutes after intensive care unit (ICU) connection to active heated humidification. The novelty lies in the sequential two-phase presentation—gradual intraoperative deterioration followed by abrupt decompensation upon inadvertent dual humidification—and its direct handover-safety implications for pediatric perioperative care.

## 2 Case presentation

A 13-month-old boy (11 kg, 75 cm, ASA I) with primary congenital glaucoma was scheduled for bilateral filtration bleb revision and goniosynechialysis under general anesthesia. An upper respiratory tract infection (URTI) 4 weeks earlier had fully resolved. Preoperative midazolam 0.5 mg was administered intravenously for anxiolysis. Standard monitoring–electrocardiography, pulse oximetry, non-invasive blood pressure, capnography–was applied. Anesthesia was induced with propofol 20 mg, sufentanil 3 μg, and cisatracurium 1 mg. Direct laryngoscopy revealed a Cormack–Lehane grade I view. However, passage of a 3.5-mm internal-diameter (ID) reinforced cuffed endotracheal tube (ETT) was obstructed at the subglottis. The trachea was ultimately intubated with a 3.0-mm ID uncuffed ETT, advanced with difficulty. Total airway instrumentation lasted approximately 3 min.

An HMEF (Emedical EM12-306) was placed between the ETT connector and the breathing-circuit Y-piece. Manufacturer specifications indicated sutability for patients 10–25 kg with tidal volumes of 60–150 mL; moisture output 32 mg H_2_O L⁻^1^, with pressure drop value of 0.03 kPa at 5 L/min, 0.05 kPa at 10 L/min, 0.10 kPa at 15 L/min, 0.15 kPa at 20 L/min. The exact internal dead-space volume was not printed on the device label, and the manufacturer-certified figure could not be retrieved despite post-event inquiry. A value of 20–30 mL, estimated from device geometry and comparable pediatric HMEFs, is used below. Ventilation was delivered on a Dräger Fabius machine in volume-controlled mode with AutoFlow. Initial settings were as follows: tidal volume (Vt) 90 mL, respiratory rate (RR) 25/min, inspiratory fraction of oxygen (FiO_2_) 0.50, inspiratory-to-expiratory ratio (I:E) 1:1.5, positive end-expiratory pressure (PEEP) 3 cmH_2_O, pressure limit 35 cmH_2_O. Auscultation revealed scattered bilateral wheezing; peak inspiratory pressure (PIP) was 25 cmH_2_O. Given the recent URTI, repeated airway manipulation, and audible wheeze, presumed bronchospasm was treated with 8 puffs of metered-dose salbutamol (100 μg/actuation) via the ETT and sevoflurane increased to an age-adjusted minimum alveolar concentration (MAC) of 2.0. PIP fell to 20 cmH_2_O; PetCO_2_ was 35–40 mmHg.

Approximately 20 min into surgery, PIP rose progressively. The circuit and ETT were inspected and found to be patent, without kinking or visible condensate. Auscultation revealed loud diffuse bilateral wheeze. Sevoflurane was increased to 2.5 MAC. The ETT withdrawn 0.5 cm to exclude endobronchial intubation, and tracheal suctioning was performed. PIP continued to rise to 35 cmH_2_O, PetCO_2_ reached 85 mmHg, and SpO_2_ fell to 95%. FiO_2_ was increased to 1.0, RR to 30/min, I:E adjusted to 1:2, and PEEP discontinued. Arterial blood gas (ABG): pH 7.038, PaCO_2_ 81.5 mmHg, PaO_2_ 105 mmHg, base excess (BE) −9 mmol/L. Severe bronchospasm was suspected. Treatment included subcutaneous epinephrine 0.01 mg/kg (0.1 mg), intravenous hydrocortisone 50 mg. Because of limited improvement, a second dose of subcutaneous epinephrine (0.1 mg) was given 20 min later, followed by intravenous magnesium sulfate 250 mg infused over 30 min. Over the next 30 min, PIP fell to 25 cmH_2_O, wheeze diminished, and PetCO_2_ fell to 45 mmHg. Repeat ABG showed: pH 7.111, PaCO_2_ 53.5 mmHg, BE −8 mmol/L. Surgery was completed uneventfully. Given the severity of the airway event, extubation was deferred. After joint consultation, the surgeon and anesthesiologist decided to transfer the patient to the ICU for further observation and management.

**Figure 1:**
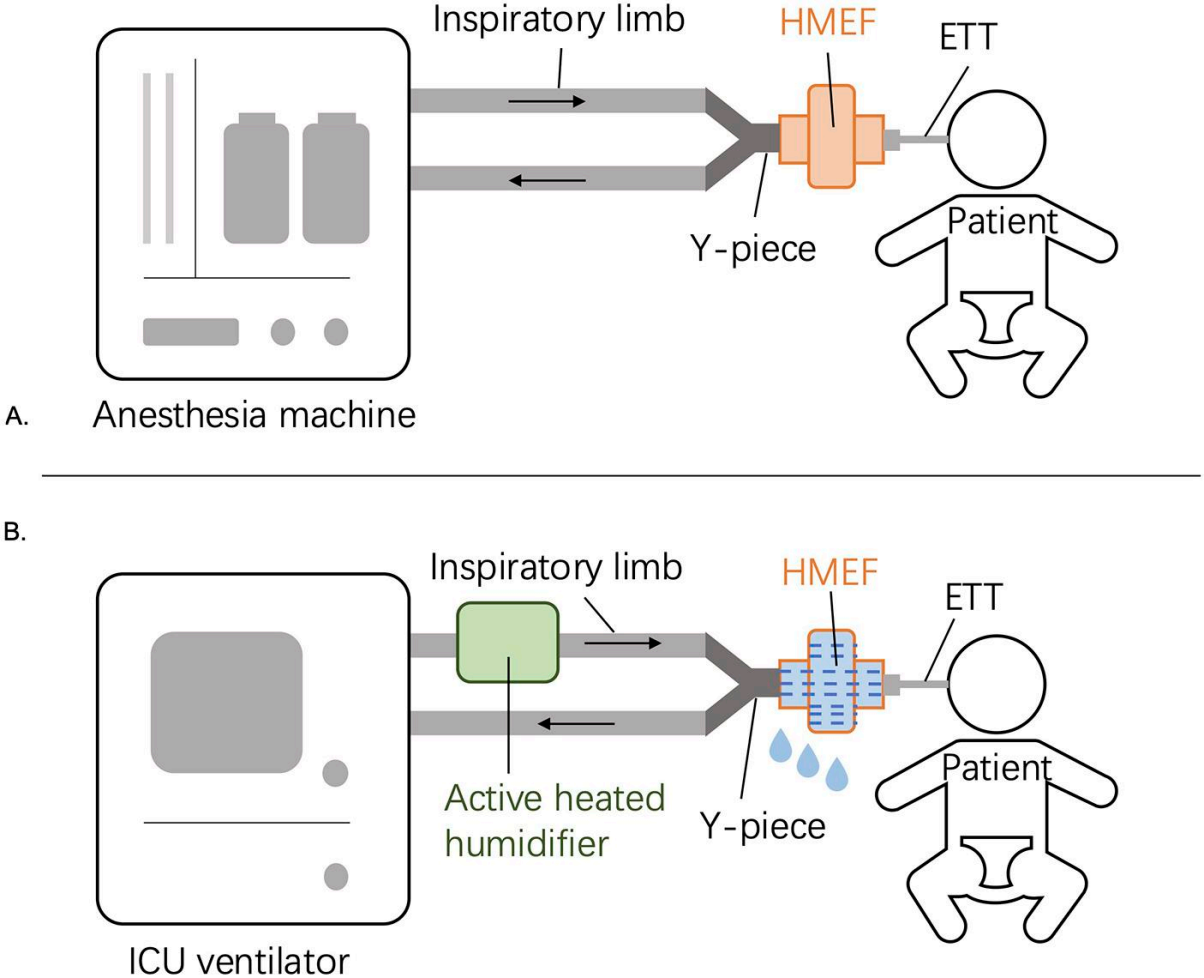
Breathing circuit configuration. **(A)** Intraoperative setup: Dräger Fabius anesthesia workstation, HMEF placed between the ETT connector and Y-piece. **(B)** ICU setup with active heated humidifier on the inspiratory limb and the intraoperative HMEF inadvertently retained distal to the Y-piece.

The patient was transported while intubated on a transport ventilator in volume-controlled mode: Vt 90 mL, RR 25/min, FiO_2_ 0.40, with no PEEP applied. The anesthesia breathing circuit and HMEF remained *in situ*. No active humidification was used during transport, and no condensate was visible in the circuit on ICU arrival. In the ICU, the same circuit—with the HMEF still in place—was connected to an ICU ventilator equipped with an active heated humidifier (chamber delivering 37 °C, 44 mg H_2_O L⁻^1^). ICU ventilator settings were: volume-controlled Vt 90 mL, RR 25/min, FiO_2_ 0.40, PEEP 5 cmH_2_O. Within approximately 10 min, PIP abruptly rose to 40 cmH_2_O and delivered Vt fell to 50 mL (≈4.5 mL/kg), while SpO_2_ remained 99%–100%. ABG results were: pH 6.916, PaCO_2_ 119.5 mmHg, BE −10.8 mmol/L, PaO_2_ 171.4 mmHg. Diffuse wheeze recurred. Inspection now revealed that the previously dry circuit had filled with condensate and that the HMEF was fully saturated. The HMEF was removed immediately. PIP fell to 20 cmH_2_O, Vt recovered to 80 mL, and wheeze resolved. ABG obtained 30 min later showed: pH 7.235, PaCO_2_ 49.9 mmHg, BE −6.8 mmol/L. The perioperative timeline is summarized in Table 1; circuit configurations in Figure 1.

**Table 1:** Timeline of key perioperative events.

Time	Event/intervention	Key parameters
T0 (intubation)	3.5-mm cuffed ETT failed; 3.0-mm uncuffed ETT placed; HMEF inserted	Cormack–Lehane I; airway instrumentation ∼3 min
T0 + 5 min	Scattered wheeze; salbutamol × 8 puffs, sevoflurane 2 MAC	PIP 25→20 cmH_2_O; PetCO_2_ 35–40 mmHg
T0 + ∼20 min	Progressive PIP rise; ETT withdrawn 0.5 cm; suctioning; FiO_2_ 1.0, RR 30/min, I:E 1:2, PEEP off	PIP 35 cmH_2_O; PetCO_2_ 85 mmHg; SpO_2_ 95%
T0 + ∼25 min	ABG #1; SC epinephrine 0.1 mg × 2, hydrocortisone 50 mg, MgSO₄ 250 mg	pH 7.038; PaCO_2_ 81.5 mmHg; BE −9 mmol/L
T0 + ∼55 min	Improvement; ABG #2	PIP 25 cmH_2_O; PetCO_2_ 45 mmHg; pH 7.111; PaCO_2_ 53.5 mmHg
ICU transfer	Transport ventilator (Vt 90 mL, RR 25/min); no active humidification; circuit/HMEF retained	No condensate on arrival
ICU + ∼10 min	Connection to ICU ventilator with active heated humidifier; abrupt deterioration	PIP 40 cmH_2_O; Vt 50 mL; SpO_2_ 99%–100%
ICU + ∼15 min	ABG #3; HMEF fully saturated; HMEF removed	pH 6.916; PaCO_2_ 119.5 mmHg; BE −10.8 mmol/L
ICU + ∼45 min	ABG #4 after HMEF removal	PIP 20 cmH_2_O; Vt 80 mL; pH 7.235; PaCO_2_ 49.9 mmHg

The child was extubated on postoperative day 2 without post-extubation stridor. Follow-up revealed coarse bilateral breath sounds with intermittent cough; a chest CT was recommended to exclude subglottic narrowing but declined by the family. He was discharged in stable condition on postoperative day 5. Written informed consent for publication was obtained from the parents; according to local institutional policy, formal ethics approval was waived for this retrospective single-case report.

## 3 Discussion

### 3.1 A mixed event with two mechanistically distinct phases

The perioperative course comprised three phases (Table 2). In Phase I (post-intubation), bronchospasm was clinically plausible given recent URTI, difficult and repeated airway instrumentation, and audible bilateral wheeze. These are recognized risk factors for perioperative respiratory adverse events, including bronchospasm, in children (7–10). The prompt PIP response to salbutamol and increased sevoflurane supports this attribution. Non-bronchospasm contributors to increased resistance likely coexisted. An ETT smaller than ideal for the airway and mucosal edema from repeated instrumentation each raise airway resistance by Poiseuille's fourth-power law, and would act synergistically with bronchoconstriction and HMEF flow resistance. Ultrasound studies suggest that subglottic diameter in children aged around 1 year is typically in the range of approximately 5.5–6.0 mm (11); the initial 3.5-mm reinforced cuffed ETT (outer diameter 5.3 mm) should theoretically have passed, so unrecognized congenital subglottic narrowing cannot be excluded, although the family declined CT evaluation.

**Table 2:** The three phases of the patient's course.

Phase	Clinical setting	Likely dominant mechanism	Evidence
Phase I	Post-intubation (OR)	Bronchospasm (± small-ETT resistance, airway edema)	URTI, repeated airway manipulation, wheeze; prompt PIP response to salbutamol + sevoflurane
Phase II	Intraoperative escalation (OR)	Mixed: bronchospasm + HMEF dead space/resistance	Progressive PIP/PaCO_2_ despite escalating therapy; reversed PetCO_2_ > PaCO_2_ gradient
Phase III	ICU post-transfer	HMEF saturation with active humidification	Abrupt deterioration within 10 min of humidifier connection; saturated HMEF; complete reversal on removal

ABG, arterial blood gas; ASA, American Society of Anesthesiologists; BE, base excess; ETT, endotracheal tube; FiO_2_, inspiratory fraction of oxygen; HME, heat and moisture exchanger; HMEF, heat and moisture exchanger filter; ICU, intensive care unit; I:E, inspiratory-to-expiratory ratio; MAC, minimum alveolar concentration; OR, operating room; PaCO_2_, arterial partial pressure of carbon dioxide; PEEP, positive end-expiratory pressure; PetCO_2_, end-tidal partial pressure of carbon dioxide; PIP, peak inspiratory pressure; RR, respiratory rate; URTI, upper respiratory tract infection; V/Q, ventilation-perfusion; Vt, tidal volume.

In Phase II (intraoperative escalation), PIP rose progressively to 35 cmH_2_O and PaCO_2_ to 81.5 mmHg despite escalating bronchodilator and anti-inflammatory therapy. Bronchospasm alone is unlikely to explain the magnitude and time course. The HMEF added 20–30 mL of apparatus dead space to an ≈90 mL tidal volume—22%–33% of each breath—and its flow-dependent resistance rises with moisture uptake. Kwon (2) and Pearsall (3) have shown that such apparatus loads disproportionately raise PaCO_2_ in small children. The reversed capnographic gradient (PetCO_2_ 85 > PaCO_2_ 81.5 mmHg) is biologically implausible without apparatus-related rebreathing, because alveolar dead space and V/Q heterogeneity make PaCO_2_ normally 2–5 mmHg higher than PetCO_2_. The most likely explanation is that the sampling port was proximal to the HMEF (toward the Y-piece) and therefore sampled a mixture of alveolar gas and CO_2_-enriched gas resident within the HMEF's 20–30 mL internal volume. Bhagwat et al. demonstrated precisely this phenomenon using dual-site capnography in a pediatric patient with HMEF-related hypercapnia, showing markedly disparate EtCO_2_ readings across the filter (12). In hindsight, this reversed gradient was a missed early clue.

In Phase III (ICU), four lines of evidence make HMEF failure the dominant mechanism. (i) Tempo: PaCO_2_ rose from 53.5 to 119.5 mmHg in ≈10 min without new airway stimulation—faster than typical bronchospasm evolves. (ii) Preserved oxygenation (SpO_2_ 99%–100%) despite severe hypercapnia favored a predominantly ventilatory rather than oxygenation-limiting mechanism. (iii) Wheeze resolved immediately on HMEF removal, inconsistent with bronchodilator kinetics (5–15 min onset); turbulent flow across a water-laden filter can generate “filter pseudo-wheeze”. (iv) The mechanism is consistent with Doyle et al. (6): when HME and heated humidifier run in series, saturated gas condenses on the cooler HMEF, reducing effective cross-section and raising resistance. In infants, smaller tidal volumes and a higher circuit surface-to-volume ratio likely accelerated saturation from hours to minutes. The abrupt reversal after HMEF removal is the strongest single causal link reported here.

### 3.2 Novelty and limitations

Lee et al. (4) described intraoperative pediatric hypercapnia related to HMEF dead space; Dewasurendra et al. (5) described rising PIP from HME obstruction in critically ill adults; Bhagwat et al. (12) showed dual-site capnographic gradients across an HMEF in a pediatric patient; and Doyle et al. (6) documented ventilator circuit occlusion from concurrent HME and heated humidifier use in a bench model. Our report adds three novel elements. First, it documents a sequential two-phase pediatric course in which gradual intraoperative hypercapnia was followed by fulminant decompensation minutes after ICU ventilator connection—a pattern that, to our knowledge, has been infrequently described in pediatric reports. Second, it provides bedside clinical, mechanical, and blood-gas evidence of HMEF–active-humidifier incompatibility in an infant, extending Doyle's adult bench observations to a real-time pediatric event. Third, it reframes perioperative handover itself as the failure point, highlighting shared responsibility between anesthesiology and surgical teams in equipment-transition safety.

We did not measure HMEF resistance or weight change, did not perform dual-site peri-filter capnography, and did not quantify delivered humidity or circuit condensate during Phase II. Had we recorded pre- and post-event filter weights, in-line resistance (pressure drop at a fixed flow before vs. after saturation), or differential capnography across the filter, we could have quantitatively partitioned HMEF- versus bronchospasm-mediated contributions in Phase II, confirmed apparatus rebreathing as the source of the reversed PetCO_2_/PaCO_2_ gradient, and reconstructed saturation kinetics. A controlled brief removal-and-retrial of the HMEF during Phase II was not performed; such a maneuver might have both confirmed the mechanism and shortened the event. The Phase II causal attribution therefore remains partly inferential, whereas the Phase III attribution is strongly supported by the immediate and sustained reversal after HMEF removal.

### 3.3 Lessons learned and proposed safeguards

Despite escalating bronchodilators, steroids, magnesium, and a second epinephrine dose, the HMEF was not inspected until after the ICU decompensation. Three biases contributed: anchoring to the initial bronchospasm attribution, availability bias favoring a familiar diagnosis over a rare equipment failure, and the invisibility of the HMEF as a “standard circuit component” that was not routinely considered during troubleshooting. Pediatric algorithms for rising PIP and unexplained hypercapnia should explicitly include filter patency, saturation, and dead-space assessment alongside tube, circuit, and patient factors—ideally as a forced cognitive step after two failed escalations of bronchodilator therapy.

The decision to transfer a postoperative pediatric patient intubated to the ICU is shared between surgeon and anesthesiologist. The HMEF—appropriately placed for intraoperative use—became dangerous once connected to active humidification in the ICU, so equipment transition is itself a perioperative safety step. We suggest incorporating an explicit breathing-circuit safety check during OR-to-ICU transfer in pediatric patients, particularly when downstream active humidification is anticipated. This simple procedural safeguard may reduce transfer-related equipment errors.

**Figure 2:**
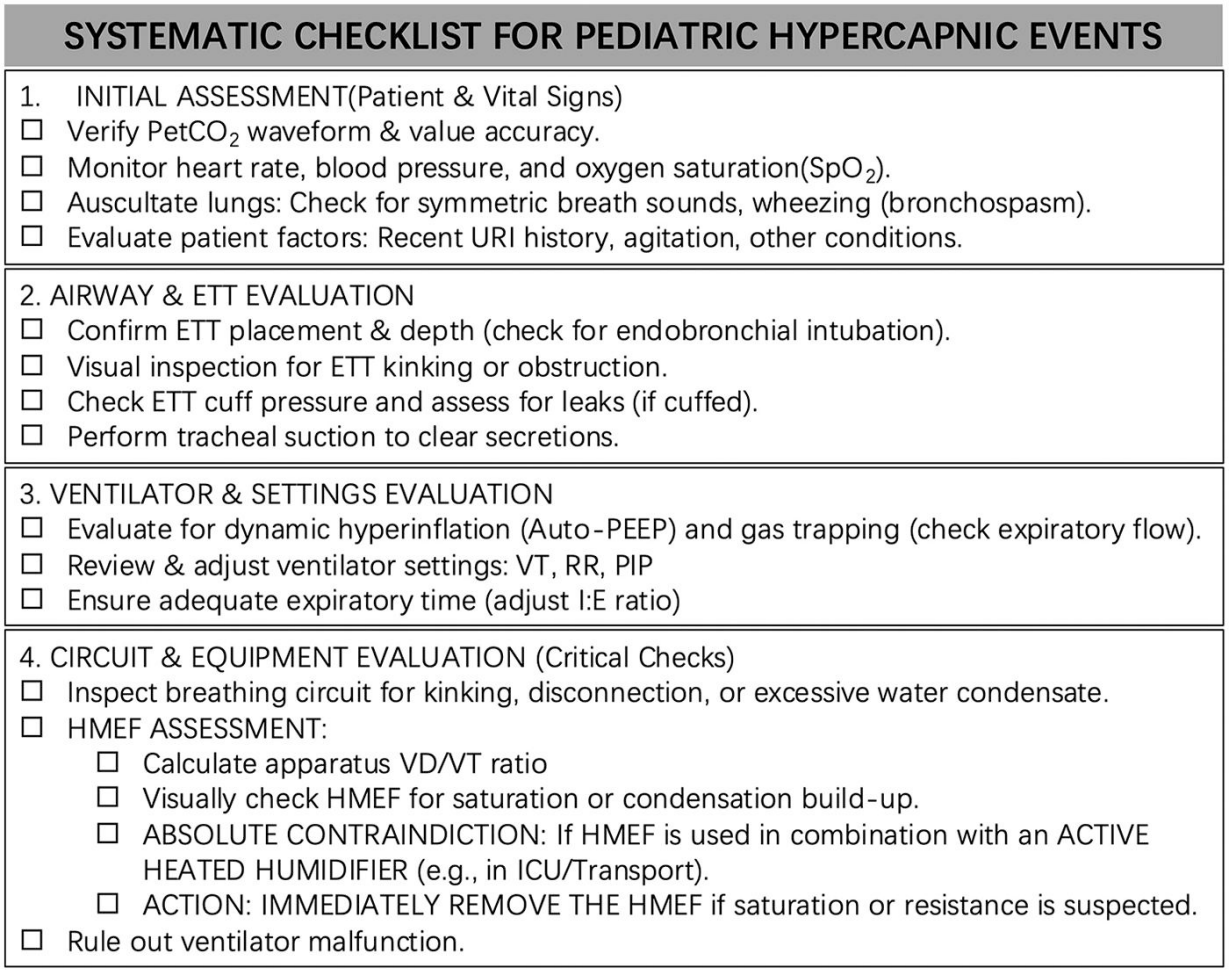
Proposed pediatric checklist for unexplained hypercapnia and rising PIP, including explicit assessment of HMEF patency, dead space, saturation, and humidifier compatibility.

Following reflection on this case, we propose the following actionable clinical recommendations: (i) For ventilated infants <15 kg with Vt <100 mL, treat HMEF dead space and resistance as active ventilatory parameters, not passive consumables; document the manufacturer's internal volume and pressure-drop specifications on the anesthesia record. (ii) In pediatric patients with tracheal intubation who develop unexplained hypercapnia, we recommend initial evaluation using a structured checklist (Figure 2). If PaCO_2_ rises disproportionately to bronchodilator response, or if PetCO_2_ approaches or exceeds PaCO_2_, inspect the HMEF, consider a brief controlled removal trial, and reassess. (iii) Before every OR-to-ICU transfer, explicitly remove the HMEF if an active humidifier will be used downstream; alternatively, replace with a new HMEF and suspend active humidification. Following 76 patient safety incidents in England involving simultaneous HME and heated humidifier use, NHS England warned against the simultaneous use of HME and heated humidification and recommended local safeguards to prevent this error (13). (iv) Consider engineered safeguards such as thermal-indicator HME labels [e.g., Humidicare, a temperature-dependent HME warning device that visually alerts clinicians when placed in a heated humidifier circuit (14)]. (v) Consider standardized dual-site or peri-filter capnography during escalating pediatric hypercapnia. (vi) Incorporate a mandatory equipment-review step into pediatric bronchospasm algorithms after failed first-line therapy.

## 4 Conclusion

In infants and small children, apparent refractory bronchospasm may coexist with, mask, or be amplified by HMEF-related ventilatory impairment. The strongest evidence in this case arose after ICU transfer, when retention of the intraoperative HMEF during initiation of active heated humidification produced abrupt ventilatory failure that reversed within minutes of HMEF removal. Pediatric perioperative teams should treat the HMEF as an active ventilatory element—verifying dead space, saturation status, and humidifier compatibility whenever hypercapnia, rising PIP, or incomplete bronchodilator response occurs, and especially at every OR-to-ICU handover.

## Data Availability

The original contributions presented in the study are included in the article/Supplementary Material, further inquiries can be directed to the corresponding author.
