# Supplementary material for: Ventilatory failure following active humidification of a retained HMEF in an intubated infant: a case report
Source: Front Surg. 2026 May 13;13:1837176. doi: 10.3389/fsurg.2026.1837176 (PMC13212268; doi:10.3389/fsurg.2026.1837176)
Supplement: Supplementary file 1 [file Table1.docx]

**CARE Checklist for Case Reports**

*Supplementary Material — Completed CARE Checklist (2013)*

**Manuscript title:** Apparent Refractory Bronchospasm Unmasking Sequential HMEF-Related Ventilatory Failure in an Infant During Transfer From the Operating Room to the ICU: A Case Report

**Authors:** Hengjing Zou, Guangyi Lai, Yanping Lu, Shan Ou

**Journal:** Frontiers in Surgery

*Please indicate in which section each item has been reported in your manuscript. The checklist follows the CARE (CAse REport) guidelines (Riley et al., J Clin Epidemiol, 2017).*

| **Topic** | **Item** | **Checklist Item Description** | **Reported on** | **Location in Manuscript** |
| --- | --- | --- | --- | --- |
| **Title** | 1 | The diagnosis or intervention of primary focus followed by the words "case report". | Page 1, Line 3-6 | Title includes "HMEF-related ventilatory failure" and ends with "a case report" |
| **Keywords** | 2 | 2 to 5 key words that identify diagnoses or interventions in this case report, including "case report". | Page 1, Line 11-12 | 5 keywords listed including HEMF, bronchospasm, hypercapnia, dead space, humidification |
| **Abstract** | 3a | Introduction: What is unique about this case and what does it add to the scientific literature? | Page 1, Line 14-19 | Background section of structured abstract describes HMEF mimicking bronchospasm |
|  | 3b | The patient’s main concerns and important clinical findings. | Page 1, Line 20-22 | Case presentation section: wheezing, rising airway pressure, severe hypercapnia |
|  | 3c | The primary diagnoses, interventions, and outcomes. | Page 1, Line 23-29 | Describes bronchospasm treatment, HMEF removal, and rapid improvement |
|  | 3d | Conclusion: What are the "take-away" lessons? | Page 1-2, Line 30-35 | Conclusions section: mixed event; prompt HMEF assessment essential in infants |
| **Introduction** | 4 | Briefly summarize why this case is unique with medical literature references. | Page 2, Line 37-57 | Introduction cites literature on HMEF dead space (refs 1–6) and explains diagnostic challenge |
| **Patient Information** | 5a | De-identified demographic and other patient-specific information. | Page 2, Line 59-64 | 13-month-old boy, 11 kg, 75 cm, primary congenital glaucoma, ASA I |
|  | 5b | Primary concerns and symptoms of the patient. | Page 3, Line 81-83 | Bilateral scattered wheezing after intubation; rising airway pressures |
|  | 5c | Medical, family, and psychosocial history including relevant genetic information. | Page 2, Line 61-63 | URI 4 weeks prior; otherwise unremarkable; no relevant family/genetic history noted |
|  | 5d | Relevant past interventions with outcomes. | Page 2, Line 62 | No relevant prior interventions; asymptomatic on day of surgery |
| **Clinical Findings** | 6 | Describe significant physical examination (PE) and important clinical findings. | Page 3, Line 82, 91-96 | Bilateral wheezing; Ppeak 25→35 cmH₂O; PetCO₂ up to 85 mmHg; SpO₂ 95% |
| **Timeline** | 7 | Historical and current information from this episode of care organized as a timeline. | Page 8, Table 1 | Table 1 provides complete timeline: post-intubation → intraop deterioration → treatment → ICU event → HMEF removal |
| **Diagnostic Assessment** | 8a | Diagnostic methods (PE, laboratory testing, imaging, surveys). | Page 3, Line 75-104 | Auscultation, ABG analysis (multiple), circuit inspection, HMEF examination |
|  | 8b | Diagnostic challenges. | Page 3-4, Line 89-123 | HMEF-related ventilatory failure mimicked bronchospasm; overlap of two mechanisms |
|  | 8c | Diagnosis (including other diagnoses considered). | Page 3-4, Line 89-123 | Mixed mechanism: coexisting bronchospasm + HMEF-related ventilatory impairment; isolated bronchospasm initially presumed |
|  | 8d | Prognostic characteristics when applicable. | N/A | Not applicable — acute perioperative event |
| **Therapeutic Intervention** | 9a | Types of intervention (pharmacologic, surgical, preventive). | Page 3, Line 84, 93-94, 100-104 | Salbutamol 8 puffs, sevoflurane 2→2.5 MAC, SC epinephrine ×2, IV hydrocortisone, IV MgSO₄, FiO₂ 100%, HMEF removal |
|  | 9b | Administration of intervention (dosage, strength, duration). | Page 3, Line 100-104 | Epinephrine 0.1 mg SC ×2 (20 min apart); hydrocortisone 50 mg IV; MgSO₄ 250 mg IV over 30 min |
|  | 9c | Changes in therapeutic interventions with explanations. | Page 3-4, Line 84-122 | Escalation from salbutamol → deepened anesthesia → epinephrine/steroids/MgSO₄ due to incomplete response; ultimately HMEF removal |
| **Follow-up and Outcomes** | 10a | Clinician- and patient-assessed outcomes when available. | Page4, Line 119-126 | After HMEF removal: PIP 20 cmH₂O, VT 80 mL, wheeze resolved; ABG improved; extubated POD2; discharged stable |
|  | 10b | Important follow-up diagnostic and other test results. | Page 3-4, Line 116-122 | Serial ABGs documented: pH 6.916 → 7.235 (30 min post-removal); no post-extubation stridor |
|  | 10c | Intervention adherence and tolerability. | Page 4, Line 124-126 | Tolerated well; successfully extubated POD2; discharged after 3 days observation |
|  | 10d | Adverse and unanticipated events. | Page 3, Line 110-117 | ICU deterioration (pH 6.916, PaCO₂ 119.5 mmHg) due to saturated HMEF + active humidification — an unanticipated event |
| **Discussion** | 11a | Strengths and limitations in your approach to this case. | Page 5, Line 184-195 | Limitation: HMEF saturation degree not quantified intraop; no CT imaging for airway anatomy |
|  | 11b | Discussion of the relevant medical literature. | Page 4-5, Line 131-183 | Cites Kwon (2), Pearsall (3), Lee (4), Dewasurendra (5), Doyle (6), Khara (7), von Ungern-Sternberg (9) |
|  | 11c | The rationale for your conclusions. | Page 4-5, Line 157-167 | ICU rapid reversal after HMEF removal provides strong causal evidence; prior literature supports mechanism |
|  | 11d | The primary "take-away" lessons (without references). | Page 5, Line 168-183 | Three lessons: review dead space in infants; evaluate equipment in refractory bronchospasm; reassess circuit during transfer |
| **Patient Perspective** | 12 | The patient should share their perspective on the treatment(s) they received. | N/A | Not obtainable — patient is a 13-month-old infant |
| **Informed Consent** | 13 | The patient should give informed consent. | Page 6, Line 211-213 | Written informed consent for publication obtained from the patient’s parents |
